# Overview of the Lives Saved Tool (*LiST*)

**DOI:** 10.1186/1471-2458-13-S3-S1

**Published:** 2013-09-17

**Authors:** Neff Walker, Yvonne Tam, Ingrid K Friberg

**Affiliations:** 1 Institute for International Programs and Department of International Health, Bloomberg School of Public Health, Johns Hopkins University, Baltimore, MD, USA

## Abstract

This paper provides an overview of the historical development and current status of the Lives Saved Tool (LiST). The paper provides a general explanation of the modeling approach used in the model with links to web sites and other articles with more details. It also details the development process in developing both the model structure as well as the assumptions used in the model. The paper provides information about how LiST has been and is currently being used by various organizations and within national health programs. We also provide a review of the work that has been done to try to validate the outputs of the model.

## Background and history

The ***Lives Saved Tool* (*LiST***) is modelling software that has been in use the past 10 years. The initial version of the software was created as part of the work for the Child Survival Series published in *The Lancet* in 2003 [1]. The original purpose of the tool was to estimate the impact that scaling up community-based interventions would have on under-five mortality [2]. From this initial starting point, the software was updated to include an expanded set of interventions focused more on facility-based care, with the additional impacts being primarily on neonatal mortality [3,4]. The model was then adapted to handle populations and cohorts in an improved version that included wasting and stunting as risk factors, added to support the *Lancet* Nutrition Series in 2008 [5]. During this period, further development and maintenance of the tool were continued as part of the work of the Child Health and Epidemiology Reference Group (CHERG) funded by the Bill & Melinda Gates Foundation.

At this point, *LiST* was shifted into the public domain by making the program freely available as part of the Spectrum Policy Modeling System software package (Spectrum). This arrangement not only offered users the advantages of the *DemProj* module to make demographic projections, it also incorporated the *AIDS Impact Module* (*AIM*) to estimate the impact of interventions affecting HIV/AIDS [6]. In more recent versions, *LiST* has been expanded further to estimate the impact of interventions on birth outcomes and stillbirths [7], maternal mortality, and pneumonia and diarrhea incidence [8], in addition to neonatal and child mortality.

## How *LiST* is used

*LiST* was initially conceived and has been extensively used to estimate the impact of scaling up interventions on mortality as a way to quantify the potential effectiveness of an intervention, to stress the need for refocusing priorities, or to set targets at a global level [2-6]. These types of analyses, conducted using a large set of countries, often capture headlines and are used by global advocacy groups.

Use of *LiST* in developing countries to guide the strategic planning process is another valuable way in which the modeling tool is being used [9]. Policymakers or national-level stakeholders may use *LiST* to develop multiple scale-up scenarios in order to compare the impact on mortality due to various combinations of proven interventions. For example, if a country has decided to roll out community health workers who will provide broader access to treatment for malaria, pneumonia and diarrhea, one can use LiST to estimate the change in coverage of these interventions over time and then estimate their combined impact on mortality. Alternatively, one can develop a second scenario where these workers are also trained to provide education about breastfeeding to young mothers. These estimates of impact of various packages of grouped interventions can then be evaluated in conjunction with cost and feasibility considerations to decide which strategy should be implemented.

A third way that *LiST* has been used is to evaluate the impact of large-scale programs that aim to scale up health services for mothers and children. In this type of analysis, *LiST* can be used to translate measured coverage changes into anticipated or projected estimates of mortality reduction [10]. Retrospectively, groups have also used *LiST* to understand and help attribute the causes of measured or observed declines in mortality to specific activities. In a recent case study in Niger, *LiST* was applied to assess the contribution of different interventions to observed reductions in child mortality [11].

## Theoretical approach and basic modeling structure of *LiST*

*LiST* has been characterized as a linear, mathematical model that is deterministic [12]. It describes fixed relationships between inputs and outputs, and the tool will produce the same outputs each time the model is run with identical inputs. For *LiST*, the primary inputs are coverage of interventions and the outputs include changes in population-level risk factors (e.g., wasting or stunting rates, birth outcomes such as prematurity or size at birth) or cause-specific mortality (e.g., neonatal or child (1-59 months), maternal mortality and stillbirths). The relationship between changes in inputs (intervention coverage) and one or more outputs is specified in terms of the effectiveness of the intervention for reducing the probability of that outcome. Outcomes of interest include either cause-specific mortality or a risk factor for mortality (e.g., intra-uterine growth restriction or stunting). The overarching assumption in *LiST* is that mortality rates and the cause of death structure will not change dynamically, and that any differences will be solely in response to changes in intervention coverage. The model assumes that changes in distal variables such as increases in per capita income or higher levels of maternal education will affect mortality by increasing coverage of interventions or reducing risk factors.

Currently, there are approximately 70 separate maternal and child health interventions within *LiST*. These evidence-based interventions have been demonstrated to reduce stillbirths, neonatal deaths, deaths among children aged 1-59 months, maternal mortality or risk factors. Interventions can be linked to multiple outcomes, with some interventions linked to multiple causes of death and risk factors. The key feature of *LiST* is that it allows one to look at the impact of scaling up coverage of multiple interventions simultaneously, and does not assess only a single intervention or single cause as is the case for many natural history models.

The model has several structural features that must be considered in order to appropriately estimate how scaling up coverage of multiple interventions and changing risk factors will impact mortality. First, the effectiveness or efficacy of an intervention must be described in terms of reductions in cause-specific mortality rather than overall mortality. With cause-specific estimates of efficacy, the combined impact of interventions can be computed. Within *LiST*, the efficacy of an intervention is defined in terms of the reduction of a cause of death or risk factor. The calculation of impact is simple when considering only a single intervention, because the change in coverage times the efficacy of the intervention is applied to the level of cause-specific mortality. For example, there may be 10,000 diarrhea deaths in children aged 1-59 months, and the proposed intervention is the introduction of a new vaccine that would be 50% effective in reducing diarrhea mortality. If coverage reaches 50%, diarrhea mortality among children would be reduced to 7,500 (=10,000 – (10,000 *0.5 *0.5)). If a second or a third intervention is added, the same approach is followed, although the impact of the additional diarrhea intervention(s) would be applied to the residual diarrhea deaths. Following the previous example, if the second new diarrhea intervention is also 50% effective and coverage reaches 50%, diarrhea mortality would fall to 5,625 (=7,500 – (7,500 *0.5 *0.5). By using cause-specific efficacy and applying each intervention to the residual deaths remaining after the previous intervention, *LiST* ensures that double counting is avoided and the potential impact of multiple interventions is not erroneously inflated.

## Age structure within *LiST*

*LiST* has a fairly simple age structure that serves as a theoretical cohort. The age periods used in *LiST* include pregnancy (among women aged 15-49), 0-1, 1-5, 6-11, 12-23 and 24-59 months for children. Within the model, impact in one age period has a cascading effect on what happens in the next period. For example, if interventions that have an impact on neonatal mortality are scaled up, more children would be expected to survive that period and will subsequently be exposed to the risk of death during the 1-59 month period. Therefore, the number of deaths in this age group will increase even though the rate of mortality will remain the same.

For maternal mortality, stillbirths, neonatal, and child (1-59 months) mortality, the cause of death structure is fixed in the base year. An appropriate mortality rate is applied that corresponds to the relevant age period; within the 1-59 month period, rates have been adjusted to reflect the higher risk of child mortality at younger ages. Maternal and child interventions specified within *LiST* often impact one or more age groups or types of mortality (i.e. maternal and neonatal mortality).

## Links to other modules in Spectrum

*LiST* runs as a linked module within Spectrum and is currently linked directly to three additional modules. A required linkage is between *LiST* and the demographic module, *DemProj*. *DemProj* is a fully functioning demographic package that allows users to define populations via inputs including age-specific fertility, migration, population structure by age and sex, and other factors. Spectrum houses a database of the most recent population projections from the United Nations Population Division for 193 countries and regions [13]. The design of this integrated system allows *LiST* users to select a country, base year, and end year, and *LiST* automatically loads in the population projection for that time period. Users can rely on this as the population projection to calculate the number of deaths by age group, but options exist to update or alter these figures within *DemProj* if needed.

*FamPlan* is the second module within Spectrum linked to *LiST*. *FamPlan* was developed to estimate the impact that scaling up family planning would have on fertility. LiST automatically loads data from FamPlan when a country is selected for analysis, and this link to *FamPlan* ensures that the most recent information available about family planning, contraceptive prevalence, unmet need for contraception, and the mix of contraceptive methods is used. LiST users can then create scenarios where unmet need is reduced, contraceptive use is increased, or the mix of contraceptive methods is changed. Altering these parameters in *FamPlan* results in changes to factors that are then inputs into both *DemProj* (including total fertility rates) and *LiST* (including distribution of risky births). Changes in *FamPlan* override the default fertility assumptions and assumptions about abortion within *DemProj*, resulting in adjusted inputs to *LiST*. If one scales up contraceptive prevalence to very high levels in a country with low contraceptive use, for example, the number of births would typically be expected to decrease and the number of averted under-five and maternal deaths predicted by *LiST* will also decrease.

Changes in family planning impact the *LiST* model to produce different outputs through two main pathways. First, as fertility changes, there are accompanying changes in the distribution of births by birth risk categories. Birth risk categories are defined by parity, maternal age and birth spacing. The relationship between changes in fertility and the distribution of births into these categories of risk have been defined by previous analyses [14]. This information is then linked to birth outcomes such as birth weight and prematurity within *LiST*. The second *LiST* input that relies upon estimates from *FamPlan* is the number of induced abortions. Because previous work has linked the availability of contraception and abortion, this information is used to estimate how the number of abortions changes as contraception changes. This information is used in *LiST* to adjust estimates of maternal mortality due to abortion.

The third module linked to *LiST* is the *AIDS Impact Module* (*AIM*), which is used to model the impact of changes in HIV/AIDS incidence, treatment and prevention on mortality. Developed under the auspices of UNAIDS and the UNAIDS reference group on modeling and estimates [15], this module describes the epidemic curve in terms of HIV incidence for each country. *AIM* includes coverage of interventions (e.g., anti-retroviral treatment, prevention of mother-to-child transmission) and uses this information to estimate prevalence and mortality by age and sex. Estimating the impact of interventions to reduce HIV/AIDS mortality in children is not done in *LiST*; the underlying calculations are done in *AIM* and then passed to *LiST*. When a country is selected within Spectrum, it will automatically load the most recent country-specific AIM module developed by UNAIDS and the national AIDS program [16]. As described for other modules within Spectrum, the user can override the standard AIM inputs, scale up interventions, and change the epidemic curve to develop new scenarios for the future. This module then passes the estimates of mortality due to HIV/AIDS for appropriate inclusion in the *LiST* module.

## Source of assumptions and the process to update *LiST*

Development of the ***Lives Saved Tool* (*LiST*)** has occurred under the guidance of the Child Health Epidemiology Reference Group (CHERG) of WHO and UNICEF. Along with its institutional sponsors, CHERG has developed rules of evidence to decide which interventions should be included in the model as well as how to develop the estimates of efficacy and effectiveness used in the model [17]. Although the assumptions used within *LiST* are drawn from various sources, most of the assumptions about the efficacy and effectiveness of interventions come from a series of journal supplements. To date, two supplements containing over 50 peer-reviewed articles have been published [18,19] with a third supplement now in press [20]. The set of assumptions and their sources can be found on the *LiST* website (http://www.jhsph.edu/IIP/list).

The CHERG also supports efforts to compare estimates that come from *LiST* to measured changes in intervention coverage and mortality. Several studies have compared actual or recorded changes in mortality to projected estimates of mortality change generated by *LiST* based upon different sets of interventions in various countries. One regional study compared *LiST* estimates to measured declines in neonatal mortality from community-based intervention trials conducted in South Asia [21]. Another study looked at community trials that focus on the scale up of the use of insecticide treated nets (ITNs) in sub-Saharan Africa [22]. A third study compared child mortality that was directly measured for a maternal and child health project implemented in Mozambique with *LiST*-produced estimates [23]. In all of these studies, there was close agreement between the estimates of mortality generated by *LiST* based upon coverage changes and the measured reductions observed for mortality. Additional studies presenting comparisons to *LiST* have been published in journal supplements [17-19].

## Creating a projection scenario in *LiST*

The basic process to create a projection scenario in LiST is straightforward. First, one must select a baseline year for a country (or region, district or any other area one chooses). For that baseline year, conditions in the country must be described in terms of a five broad sets of variables: mortality, exposure, risk factors, intervention coverage, and demography. For mortality, one must specify the neonatal and child (1-59 month) mortality, stillbirth rates, and maternal mortality rates, as well as the proportional causes of mortality or stillbirths. Exposure variables include factors such as exposure to *P. falciparum*, levels of deficiency for vitamin A and zinc, and the percent of the population living in poverty. Risk factors include stunting and wasting rates by age, birth outcomes, breastfeeding patterns and diarrhea and pneumonia incidence. Coverage data on interventions must be provided for all interventions in *LiST* for the baseline year. Finally, basic demographic information must be provided for *LiST* to operate, including population structure by age and sex as well as age-specific fertility. Fortunately, *LiST* (similar to the other linked Spectrum modules) can be automatically loaded with default values for 90 low- and middle-income countries for any year from 2000 to 2012, with information that is typically compiled from large population-based surveys such as Demographic and Health Surveys (DHS) or Multiple Indicator Cluster Surveys (MICS). Once the country and base year are selected, the information is automatically loaded, although the user has the option to change values if they have better data or if they would like to modify the population to reflect subnational geographic areas.

Once the baseline year is set for a country, the user can then create a projection scenario by scaling up coverage of a single or multiple interventions over a time period. For example, one could look at the impact of scaling up vitamin A supplementation from its current (hypothetical) level of coverage of 50% in 2013 to 95% coverage in 2015. One could alternatively develop a treatment scenario where scale up of coverage for the treatment of diarrhea with oral rehydration solution (ORS), antibiotics for pneumonia, and treatment of malaria with artemisinin-based combination therapy (ACT) reaches 90% by 2018 from current coverage levels.

Once a scale up scenario has been created, *LiST* then re-computes all of the inputs used in the base year for all subsequent years, based on the anticipated impact of the interventions in the scale up scenario. The levels of mortality, cause of death structure, and levels of risk factors will be recomputed and applied to the new population structure that reflects not only the changes from *DemProj* but also any changes in the intervention coverage from the *LiST* model and changes made in the *FamPlan* and *AIM* modules.

## Attribution of lives saved

A valuable output provided by the *LiST* model is the attribution of lives saved to changes in coverage of specific interventions and risk factors. When a single intervention is scaled up, attribution is simple. However, when multiple interventions are scaled up that act on the same cause of death, a set of standardized attribution rules is needed. *LiST* first attributes impact to all preventive interventions (ordered sequentially from periconception, through pregnancy, delivery, followed by the specific age groups described previously), and then attributes impact to the curative interventions, also within this sequential pattern. Thus, if both a preventive and a curative intervention are scaled up simultaneously, the full effect of the change in coverage of the preventive intervention is calculated first and attributed to the preventive intervention. Then any residual deaths averted are calculated and attributed to the curative intervention.

When there are two or more interventions in either preventive or curative categories with an impact on the same cause of death, an additional step is required for the attribution calculation. First, we compute the number of lives saved by applying all preventive interventions. The attribution is based on the proportional impact of the preventive interventions, calculated as the increase in coverage times the effectiveness of the intervention. For example, we may have two interventions for reducing pneumonia mortality, with intervention A estimated at 40% effectiveness and intervention B estimated at 50% effectiveness. Current coverage of interventions A and B is 30% and 40%, respectively. If both interventions are scaled up to 50%, then the relative impact of intervention A would be (0.5-0.3) * 0.4 = 0.08, and for intervention B it would be (0.5-0.4) * 0.5. = 0.05. The total lives saved by the scale up of these two interventions would be allocated, with 61.5% attributed to intervention A (.08/(0.08+0.05)) and 38.5% to intervention B (.05/(0.08+0.05)). This same attribution approach is followed if there are three or more preventive interventions and/or curative interventions. Additional examples of detailed calculations can be found in Winfrey et al [24].

## Linked work and future directions

The ***Lives Saved Tool*** (*LiST*) draws on available datasets to predict changes in mortality due to maternal and child health interventions, family planning activities and HIV/AIDS programs. By combining these in a consistent and transparent way within the Spectrum Policy Modeling System, *LiST* has become a powerful software tool, able to predict the potential benefits of each of these types of activities alone or together. Also as LiST and Spectrum are freely available for download and source code is available on request, there have been several groups that have linked to this work. Recently, there has been the development of the OneHealth tool (another module within Spectrum), which links to the impact analyses performed within LiST and other modules such as AIM and FamPlan [25]. OneHealth is a comprehensive costing and financial planning module that can be used by countries to develop strategic financial plans for a broad set of interventions within the areas of reproductive, maternal, neonatal and child health. LiST as well as its simpler precursors has also spawned several related pieces of software that while using the approach and assumptions from LiST have been translated into separate, standalone pieces of software. We will briefly review two of these tools below.

The marginal budgeting for bottlenecks tool (MBB) is an analytical costing and budgeting tool that helps countries develop their health plans by taking into account the most effective interventions, cost and budget marginal allocations of their implementation to health services and assess their potential impact on health coverage [26]. This tool was developed with support from UNICEF and the World Bank using the initial inputs from the modeling work of Jones and colleagues for the Lancet Child Survival series in 2003 to estimate impact. MBB is a standalone piece of software [26] and is built in excel spreadsheets. Initially there were efforts to keep MBB’s assumptions related to impact of interventions linked to those in LiST, but over time this linkage has become less clear.

A second tool is Mandate, the Maternal and Neonatal Directed Assessment of Technology project, is a framework for the global community to prioritize technology development and to estimate the impact of those technologies on maternal, fetal, and neonatal mortality [27]. This piece of software is web based and incorporated the approach and assumptions from LiST to estimate the impact of new technologies in reducing mortality for women and children. Much like MBB, the Mandate tool links the impact assumptions from LiST to bottlenecks that limit coverage of interventions. Mandate was explicitly developed to help prioritize the development of new interventions for maternal and child health.

The development of these related tools along with the widespread use of LiST by various organizations has been facilitated by the underlying collaborative approach to the development of LiST. Both the World Health Organization and UNICEF have served to guide the development of the tool through their Child Health Epidemiology Reference Group. This group also brings together a divergent set of researchers in the area of MNCH who, with limited support of project funds, provide the guidance and reviews for the assumptions used in the model. The Institute for International Programs at the Bloomberg School of Public Health, working with the Futures Institute, has been responsible for the developing and improving the model in response to both its academic partners and in response to needs to users. With the continuing support from the Bill and Melinda Gates foundation we anticipate that we will be able to continue to refine and improve LiST with the goal of supporting countries and their partners in improving programming for mothers and children.

## Competing interests

The authors declare that they have no conflict of interest.

## Declaration

The publication costs for this supplement were funded by a grant from the Bill & Melinda Gates Foundation to the US Fund for UNICEF (grant 43386 to "Promote evidence-based decision making in designing maternal, neonatal, and child health interventions in low- and middle-income countries”). The Supplement Editor is the principal investigator and lead in the development of the Lives Saved Tool (LiST), supported by grant 43386. He declares that he has no competing interests.

This article has been published as part of *BMC Public Health* Volume 13 Supplement 3, 2013: The Lives Saved Tool in 2013: new capabilities and applications. The full contents of the supplement are available online at http://www.biomedcentral.com/bmcpublichealth/supplements/13/S3.
